# Microwave-Assisted Two-Step Liquefaction of Acetone-Soluble Lignin of Silvergrass Saccharification Residue for Production of Biopolyol and Biopolyurethane

**DOI:** 10.3390/polym13091491

**Published:** 2021-05-06

**Authors:** My Ha Tran, Ju-Hyun Yu, Eun Yeol Lee

**Affiliations:** 1 Department of Chemical Engineering (Integrated Engineering), Kyung Hee University, Yongin-si 17104, Korea; tranhamy759@gmail.com; 2 Bio-based Chemistry Research Center, Advanced Convergent Chemistry Division, Korea Research Institute of Chemical Technology, P.O. Box 107, 141 Gajeong-ro, Yuseong-gu, Daejeon 305-600, Korea

**Keywords:** silvergrass, acetone-soluble lignin, microwave heating, two-step liquefaction, biopolyol, biopolyurethane

## Abstract

The application of microwave heating facilitated efficient two-step liquefaction of acetone-soluble lignin obtained from saccharification residue of *Miscanthus sacchariflorus* (silvergrass), which was prepared by enzymatic hydrolysis, to produce biopolyol with a low acid number and favorable hydroxyl number. The acetone-soluble lignin was liquefied using a crude glycerol and 1,4-butanediol solvent mixture at various solvent blending ratios, biomass loadings, acid loadings, and reaction temperatures. The optimal reaction condition was determined at a solvent blending ratio of crude glycerol to 1,4-butanediol of 1:2, 20% of biomass loading, and 1% of catalyst loading at a reaction temperature of 140 °C for 10 min. Subsequently, the optimal biopolyol was directly used for the preparation of biopolyurethane foam as a value-added product. The chemical and physical properties of biopolyurethane foams derived from acetone-soluble lignin were characterized by Fourier transform infrared spectroscopy (FT-IR), thermogravimetric analysis (TGA), and high-resolution scanning electron microscopy (HR-SEM). In addition, mechanical properties of produced biopolyurethane foams, including compressive strength and density, were also characterized to suggest their appropriate applications. The results indicated that the biopolyurethane foam can be used as a green replacement for petroleum-based polyurethane foam due to its comparable thermal properties, mechanical strength, and morphological structure.

## 1. Introduction

In terms of the energy supply, fossil fuels always play a crucial role, although their enormous utilization steadily causes numerous environmental problems. Not only global warming but also air and water pollution impose certain negative impacts on human health and the Earth [1,2,3]. Furthermore, fossil fuels are non-renewable resources and will be depleted in the next 40–50 years [4,5]. Therefore, it is very important to find alternative renewable resources for the development of a sustainable energy economy. Among the various renewable resources, biomass is currently attracting more attention due to its significant advantages such as high abundance, high availability, carbon-neutral nature, versatility, and low cost in comparison to fossil fuels [1,6,7,8]. Specifically, lignocellulosic biomass, which is a class of second-generation biomass, has emerged as a promising feedstock for various chemical industries. Many studies have revealed that lignocellulosic biomass feedstocks such as agricultural residues (crop straws, corn stover), herbaceous crops (switchgrass, silvergrass), and forestry residue have more desirable properties over other species of biomass because they can be produced quickly in large quantities and do not compete with food production [9,10,11]. Fundamentally, lignocellulosic biomass is mainly composed of cellulose (25–55%), hemicellulose (11–50%), and lignin (10–40%). Among various utilizations of lignocellulosic biomass, saccharification is one of the most widely used methods for the production of biofuels and biochemicals from lignocellulosic biomass [12]. After biomass saccharification, the remaining saccharification residue, mainly containing lignin, can be used as an abundant aromatic feedstock for different thermochemical and biological conversions to produce a variety of value-added products [13].

Among the various reported approaches to produce valuable products from low-value lignin residue, solvothermal liquefaction is one of the most common and effective processes [14,15,16]. Compared to other thermal processes, liquefaction of lignin generates a smaller set of depolymerized products under milder conditions, leading to lower energy consumption for processing. During the liquefaction, lignin is degraded and decomposed into smaller fragments with increased functionality through solvolytic reactions in the presence of a liquefying solvent and catalyst [15]. The depolymerized product mixture obtained after liquefaction, mainly consisting of phenolic oligomers with multiple hydroxyl groups per molecule, was commonly designated as biopolyol, which is an important intermediate for further preparation of biopolymers such as biopolyurethane [17,18]. Either acid or base catalysts may be used for liquefaction to promote and improve the liquefaction yield with different benefits and certain drawbacks [15]. Recently, two-step liquefaction using both acid and base catalysts has been reported to combine the advantages of both catalysts and to produce highly efficient liquefaction and biopolyol with comparable properties to those of petroleum-based polyols [14,19]. In particular, utilization of an acid catalyst resulted in a high biomass conversion; next, the base catalyst was used to treat biopolyol for better quality with low acidity, making it suitable for the sequential preparation of biopolyurethane, an extremely versatile polymer used in various applications with enormous consumption every year [14].

Recently, many studies have reported that the application of microwave energy could enhance the efficiency of various processes in indicated industries. Compared with conventional heating based on interfacial heat transfer, microwave heating provides direct and rapid heating throughout the entire volume of the reactor at the molecular level, thereby significantly increasing the reaction rate and shortening the reaction time while ensuring high liquefaction efficiency. Accordingly, the reaction time for microwave-assisted liquefaction can be dramatically reduced to 5–30 min, compared to 90–120 min for convention liquefaction [20,21,22,23,24]. Compared to liquefaction using conventional heating, microwave heating greatly accelerated the depolymerization reaction of biomass [22]. In addition, microwave heating has several advantages in improving the physical and chemical properties of the resulting products as well as fewer hazards for the environment [25].

Herein, for the first time, acetone-soluble lignin obtained from saccharification residue of *Miscanthus sacchariflorus* (silvergrass) was subjected to microwave-assisted two-step liquefaction using both an acid catalyst and base catalyst to produce high-quality biopolyol. The effects of various reaction parameters on the biomass conversion and acid and hydroxyl number of resulting biopolyols were fully examined to determine the optimal condition. Subsequently, biopolyol produced from the optimal condition was directly used for the preparation of biopolyurethane foam. Finally, synthesized biopolyurethane foams were characterized by Fourier transform infrared (FT-IR) spectroscopy, thermogravimetric analysis (TGA), high-resolution scanning electron microscopy (HR-SEM), and determination of mechanical properties.

## 2. Materials and Methods

### 2.1. Materials

Unrefined crude glycerol (CG, GS Caltex, Seoul, Korea) consisting of 80 wt% glycerol, 12 wt% water, and other impurities was used as the liquefying solvent without further purification [18]. The co-solvent, 1,4-butanediol (BD, Daejung Co., Gyeonggi-do, Korea), was used with the crude glycerol. The acid and base catalysts utilized 95% sulfuric acid (Sam-chun Co., Seoul, Korea) and sodium hydroxide beads (Daejung Co., Gyeonggi-do, Korea), respectively. For the determination of the acid and hydroxyl numbers, ethanol (Daejung Co., Gyeonggi-do, Korea), phthalic anhydride (Tokyo Chemical Industry Co., Tokyo, Japan), pyridine (Sam-chun Co., Seoul, Korea), imidazole (Daejung Co., Gyeonggi-do, Korea), and standard sodium hydroxide (0.1 N and 0.5 N, Daejung Co., Gyeonggi-do, Korea) were used. To synthesize polyurethane foam, poly [(phenyl isocyanate)-co-formaldehyde] (PMDI, Sigma-Aldrich Co., Seoul, Korea) and a preresin mix (PIUSYS, Gyeonggi-do, Korea) were used [19]. All chemicals were of analytical reagent grade without further purification.

### 2.2. Preparation of A High-Lignin Residue from Miscanthus Sacchariflorus

As described in a previous report, *Miscanthus* (*Miscanthus sacchariflorus*) was supplied by an energy crop plantation company (Iksan, Korea) [26]. A 100 kg sample of air-dried and chopped *Miscanthus* was hydrated and ground at the same time in a twin-screw mill (TEK 40 MHS, SM Platek, Seoul, Korea). To the mashed biomass, a ten-fold volume (final content to the dried biomass) of water was added and mixed vigorously. The insoluble fiber was separated as a solid cake using a filter press (Tae Young Filtration System, Yeongcheon, Korea) operated at 15 bars. The cake was broken down with a cutter mill and then hydrothermally treated at 200 °C for 10 min using a continuous high-pressure reactor, the SuPR2G (AdvanceBio Systems, Milford, OH, USA). Deionized water (five-fold weight of the pretreatment slurry) was added and the aqueous phase was again squeezed in a filter press. The solid cake of the pretreated *Miscanthus* was re-broken with a cutter mill and refined mechanically with a disc mill (Laboratory disc mill, Andritz, Muncy, PA, USA) at a gap size 0.003”. In a pair of stirred tank reactors (75 L) equipped with ribbon type impellers, the pretreated solid (6250 g as dry matter) of *Miscanthus* was added, respectively, and enzymatically hydrolyzed with 198.3 mL of Cellic CTec3 (10 FPU/g glucan) at 50 °C for 96 h. The acidity of the hydrolysates was maintained at pH 5.0 with aqueous ammonia (3%, *v*/*v*). Afterward, the hydrolysates were cooled at room temperature. Isolated soy protein (10 g, MP Biomedicals, LLC, Alsace-Champagne-Ardenne-Lorraine, France) was dispersed in deionized water (1 L) and autoclaved at 105 °C for 1 h. This aqueous soy protein was added to the *Miscanthus* hydrolysate as a coagulant for S/L (solid/liquid) separation. The pH of the hydrolysate was adjusted to 3.5 to promote the coagulation of fine particles. Then, the hydrolysate was filtered with a filter press to recover an aqueous sugar. The residue was completely dispersed with 35.6 L of deionized water and filtered again to wash the insoluble solid as a saccharification residue. The residue was lyophilized so as not to be hardened. Chemical compositions of feedstock and the saccharification residue were assessed by the laboratory analytical procedure (LAP) of NREL [27]. Monomeric sugars in the hydrolysate were quantitatively determined by HPLC (Waters binary HPLC pump, Model 1525; Waters autosampler, Model 2707; and Waters refractive index detector, Model 2414, Milford, MA, USA) with Aminex HPX-87H columns (300 × 7.8 mm^2^, Bio-Rad Laboratories, Inc., Hercules, CA, USA). The lyophilized saccharification residue (1 kg) was extracted with 10 L of acetone at room temperature three times, and acetone was removed by reduced pressure distillation. The obtained solid was washed with deionized water three times and dried at room temperature to obtain acetone-soluble lignin.

### 2.3. Microwave-Assisted Two-Step Liquefaction of Acetone-Soluble Lignin

Microwave-assisted liquefaction of acetone-soluble lignin was carried out in a 150 mL reaction beaker equipped with a mechanical stirrer and MAS-II Plus Microwave Synthesis Workstation (SINEO Microwave, Shanghai, China) under atmospheric pressure. In the first step, a preweighed amount of acetone-soluble lignin and 30 g of the solvent mixture were added into the reactor. The reactor was preheated to the desired temperature in 5 min at 500 W of microwave power and 200 rpm of constant stirring. Afterward, the acid catalyst was added in a predetermined amount and the acid-catalyzed liquefaction was continued for 10 min. After the termination of the reaction, the product was cooled to room temperature. Approximately 10% of the liquefied product was used for the determination of biomass conversion and the rest was neutralized with 1 N NaOH solution. The neutralized product was fractionated with 100 mL of 99.5% acetone and then filtered by filter paper (Whatman #4) to remove unreacted lignin. The acetone was then evaporated to obtain the biopolyol.

**Scheme 1:**
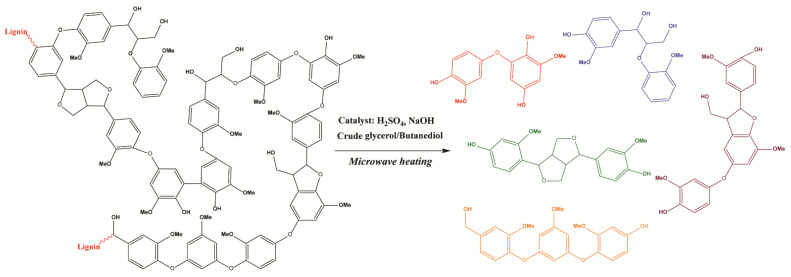
Schematic representation of lignin liquefaction to produce biopolyol.

In the second step, the obtained biopolyol was treated by a base-catalyzed medium to improve its quality for subsequent preparation of biopolyurethane. This process was conducted in the same reactor system as the first step. Biopolyol and 30 g of the solvent mixture were added to the reactor. The reactor was preheated at 120 °C in 5 min with constant stirring (200 rpm). After preheating, the base catalyst, NaOH powder, in an amount equal to 1% of the biopolyol weight was added into the reactor. The treatment process was continued for 10 min. After treatment, the biopolyol was analyzed to determine the acid number and hydroxyl number as previously described [19]. For visualization of the liquefaction process, the schematic representation of lignin liquefaction to produce biopolyol is presented in Scheme 1.

### 2.4. Determination of Biomass Conversion

Approximately 10% of the liquefied product was weighed and washed in a 250 mL centrifuge tube with 50 mL of ethanol. The centrifugation was conducted at 10,000 rpm for 15 min and the liquid phase was discarded. Another 50 mL of ethanol was added for washing and the centrifugation was repeated to ensure the removal of the biopolyol. The solid residue remaining in the tube was rinsed with distilled water and then obtained by filtration using a preweighed filter paper (Whatman #4). The filter paper with residue was dried for 12 h in a 105 °C drying oven and weighed to calculate the biomass conversion as:

(1) $$BC\;\left(\%\right)=100-\frac{\frac{W_{1}-W_{2}}{W_{3}}\times W_{4}\times 100}{W_{5}}$$

where:

BC: biomass conversion (%);

W_1_: total dry weight of the filter paper with solid residue (g);

W_2_: dry weight of the filter paper without solid residue (g);

W_3_: weight of the biopolyol used for biomass conversion determination (g);

W_4_: total weight of the biopolyol produced from the liquefaction process (g);

W_5_: weight of the acetone-soluble lignin used for liquefaction (g).

### 2.5. Preparation of Polyurethane Foam

Biopolyurethane foam was synthesized from the optimal biopolyol (obtained in the optimal condition) by a one-shot method. Three biopolyurethane foams, PUF1, PUF2, and PUF3, were prepared based on the weight ratio between biopolyol and a preresin mix of 1:9, 2:8, and 3:7, respectively. In total, a 10 g sample of biopolyol and preresin mix was prepared as polyol mixture by thoroughly mixing in a plastic cup at a speed of 900 rpm for 30 s by a mechanical stirrer. Afterward, 10 g of PMDI were added into the cup and the combination was stirred for another 30 s, and then allowed to grow at ambient temperature. After conditioning, biopolyurethane foams were cut into pieces to a standard size and characterized following the standard protocols. A polyurethane foam without the addition of biopolyol (named as neat polyurethane foam, PUF0) was also prepared for comparison.

### 2.6. Characterization of Acetone-Soluble Lignin, Biopolyol, and Polyurethane Foams

Acetone-soluble lignin, biopolyol, and all synthesized polyurethane foams were analyzed using an FT-IR spectrometer (Spectrum One System, Perkin-Elmer, Waltham, MA, USA) in KBr pellets and attenuated total reflectance (ATR) mode over a frequency range of 4000 cm^−1^ to 450 cm^−1^_._

Thermogravimetric analysis (TGA) of acetone-soluble lignin, biopolyol, and polyurethane foams was conducted using a TGA Q5000 IR (TA Instruments, New Castle, DE, USA) under a nitrogen atmosphere with a temperature range from 20 to 900 °C at a heating rate of 20 °C/min.

HR-SEM (MERLIN, Carl Zeiss, Germany) was applied to measure the cell structure and morphology of the polyurethane foams. All samples were cut into pieces, then Pt coated to provide an electrically conductive surface before scanning. Images were recorded under an accelerating voltage of 10 kV and magnification of 150.

The compressive strength of biopolyurethane foam and neat foam were measured according to ASTM D 1621. The test was performed using a universal testing machine with a load of 3 KN at a crosshead speed of 3 mm/min. The size of the specimen was 30 mm × 30 mm × 30 mm (length × width × thickness). At least five samples were used for compressive strength measurement and an average value was calculated along with the standard deviation. The density of the biopolyurethane foam was measured at 23 °C at 50% relative humidity according to ASTM D1622-03. The specimen size was 30 mm × 30 mm × 30 mm (length × width × thickness). Five specimens were used per sample and the average value was reported.

## 3. Results and Discussion

### 3.1. Composition Analysis of High-Lignin Residue from Miscanthus

**Figure 1:**
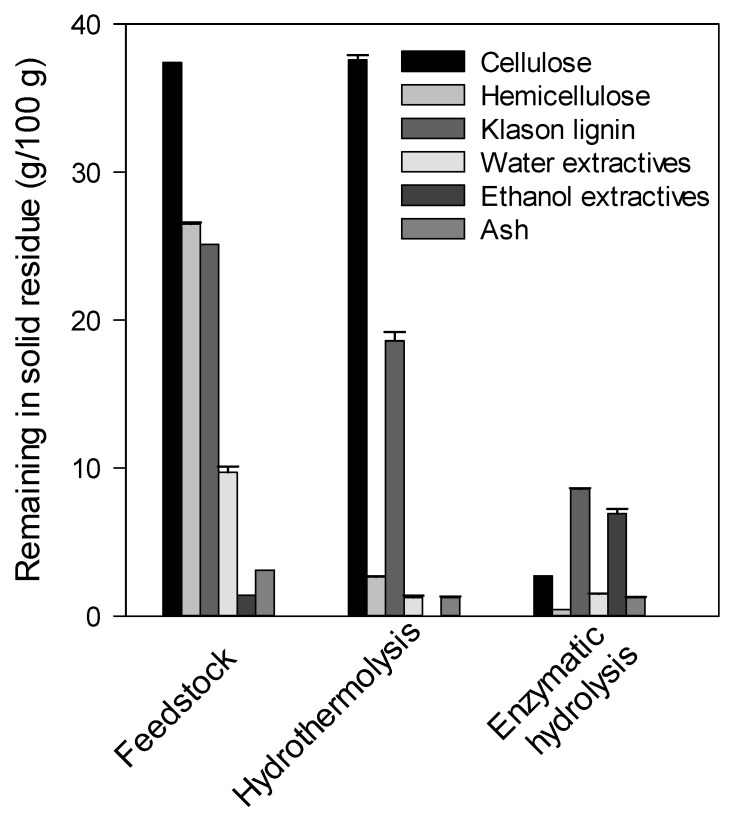
Each component remaining in dry solid during fermentable sugar production with *Miscanthus*.

The composition of the initial feedstock, pretreatment solid, and saccharification residue is presented in Table 1. Figure 1 shows each component remaining during the process. Total glucan and lignin content in 100 g of dried *Miscanthus* as a feedstock was 39.6 g (37.4 g cellulose and 2.2 g free sugar) and 25.1 g, respectively. After water extraction followed by hydrothermolysis at 200 °C for 10 min, the contents in the pretreated solid containing about 5% of the liquefied biomass were increased to 59.1 g (57.2 g cellulose and 1.9 g sugar) and 28.3 g, respectively. Not only were almost all of the hemicelluloses (90.1%) removed, but the amount of lignin was also reduced from 25.1 g to 18.5 g. During the pretreatment, 26.3% of the lignin was solubilized. Due to the hydrothermolysis, the enzymatic digestibility of cellulose reached 89.7%, and 33.3% of the saccharification substrate was obtained as an insoluble residue containing lignin. In the compositional analysis of the residue using a Soxhlet apparatus, the ethanol-soluble fraction was 31.6% and thought to be low molecular weight phenolics. The free sugar in the residue was less than 3% due to the two-step process for sugar recovery. However, most of the inorganic component in the pretreated solid was reserved in the solid residue during enzymatic saccharification. When acetone-soluble lignin was prepared from saccharification residue of silvergrass, it was a light brown dry solid. Additionally, insoluble residue of a 10-fold volume of acetone was less than 1%.

### 3.2. Optimization of Reaction Conditions for Microwave-Assisted Liquefaction of Acetone-Soluble Lignin

#### 3.2.1. Effect of Solvent Blending Ratio

**Figure 2:**
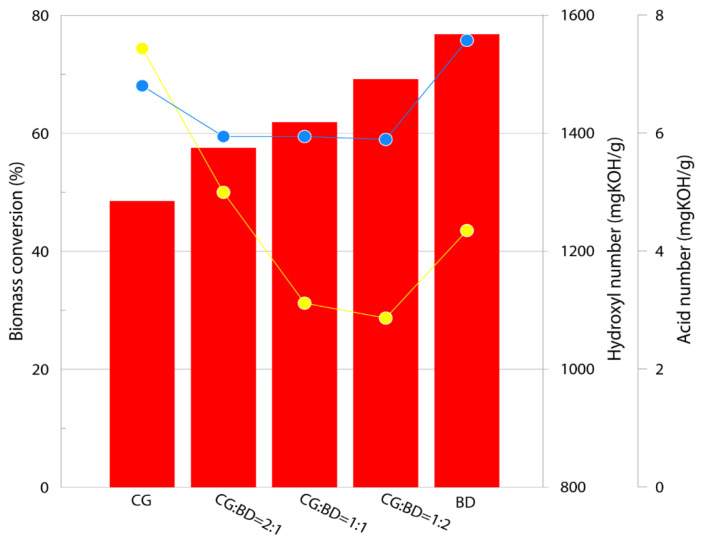
Effect of solvent blending ratio on biomass conversion and biopolyol acid number (blue line) and hydroxyl number (yellow line). Reaction condition: temperature, 120 °C; biomass loading, 10%; catalyst loading, 1%; reaction time, 10 min.

In this study, crude glycerol (CG) and 1,4-butanediol (BD) were used as the liquefying solvents. Different solvent blending ratios from 1:2 to 2:1 (CG:BD, weight ratio) and the use of CG and BD alone were examined to determine the most suitable ratio for the liquefaction of acetone-soluble lignin. The biomass conversion of acetone-soluble lignin and acid and hydroxyl numbers of the biopolyol products with different solvent ratios are presented in Figure 2. As the solvent ratio of CG to BD varied to a higher BD volume, it was observed that the biomass conversion increased from 48.52% to 76.79%. As mentioned above, unrefined crude glycerol containing a small fraction of impurities was used without purification; therefore, it might result in certain adverse effects on liquefaction, resulting in a lower conversion rate than liquefaction using BD [18]. On the other hand, CG is a low-cost byproduct of biodiesel production, which is produced in large quantities, thus the utilization of CG as a co-solvent with BD for acetone-soluble lignin liquefaction could improve the economic value of biopolyol and biopolyurethane products due to the reduced cost of raw materials. Therefore, to ensure the high biomass conversion and achieve promising techno-economic feasibility of the liquefaction process, the blending ratio of CG to BD was determined as 1:2 and subsequently used for the following experiments.

#### 3.2.2. Effect of Biomass Loading

**Figure 3:**
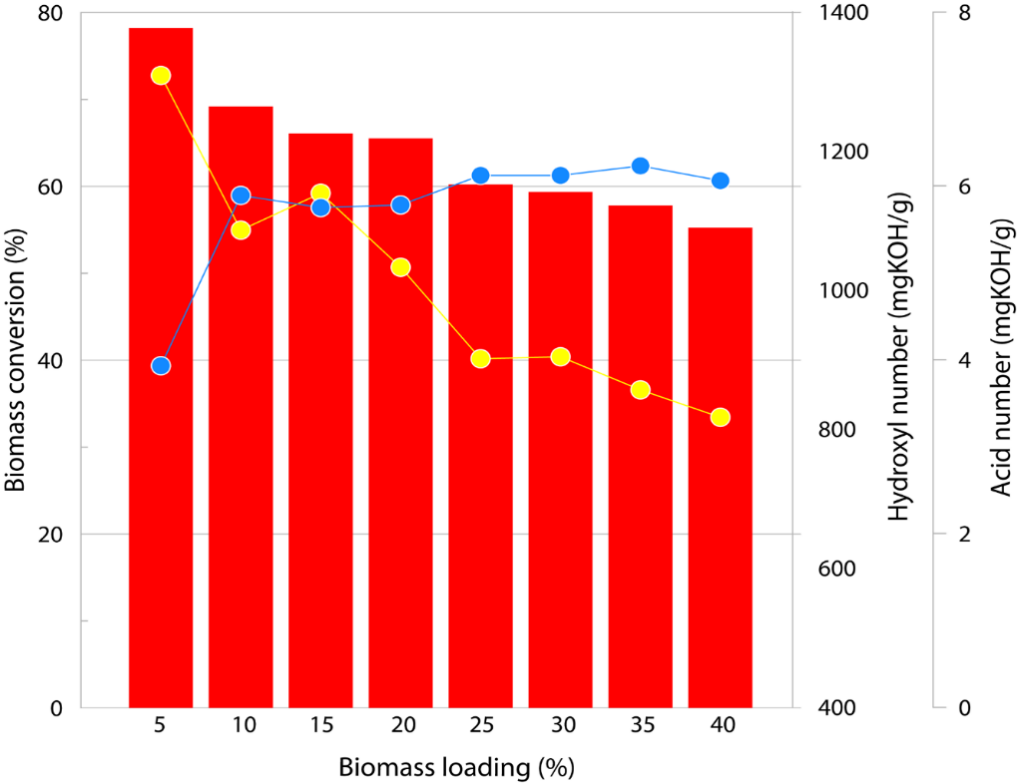
Effect of biomass loading on biomass conversion and biopolyol acid number (blue line) and hydroxyl number (yellow line). Reaction condition: solvent blending ratio, 1:2; temperature, 120 °C; catalyst loading, 1%; reaction time, 10 min.

Biomass loading is an important factor and can significantly affect the yield of the liquefaction process. Additionally, biomass loading determined based on the liquefying solvent is particularly related to the cost of processing. Therefore, biomass loading must be optimized to compromise between the maximum biomass conversion and minimum solvent amount. As shown in Figure 3, the biomass conversion gradually decreased when the biomass loading increased from 5% to 40%. The dwindling of the conversion rate is mainly due to the insufficiency of the solvent for dispersing and liquefying biomass [18,28]. As the biomass loading increased from 15% to 20%, it can be observed that the biomass conversion was probably similar, around 66%. However, a further increase in biomass loading to 25% resulted in a dramatically reduced conversion efficiency. In addition, at 20% biomass loading, the produced biopolyol also possessed a moderate hydroxyl number and low acid number, which makes it suitable for later preparation of biopolyurethane [19,29]. When considering the solvent amount used for liquefaction, 20% biomass loading was selected as the optimal biomass loading for acetone-soluble lignin liquefaction because it required a smaller amount of solvent for liquefying the same amount of lignin with a similar liquefaction yield.

#### 3.2.3. Effect of Catalyst Loading

**Figure 4:**
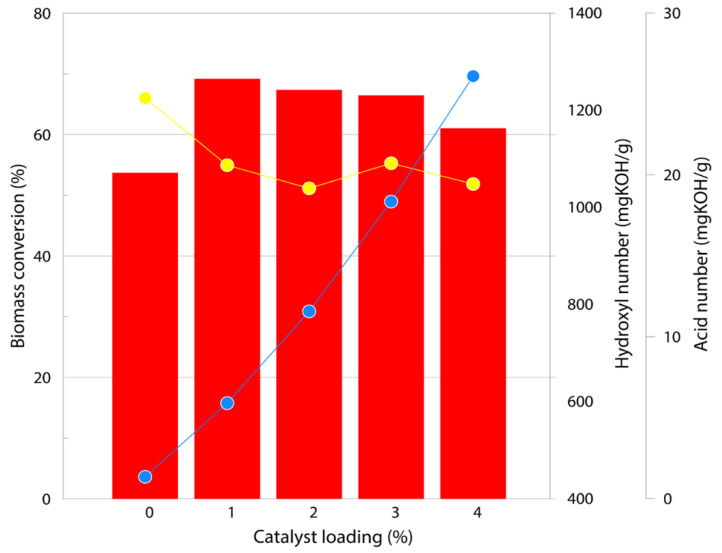
Effect of catalyst loading on biomass conversion and biopolyol acid number (blue line) and hydroxyl number (yellow line). Reaction condition: solvent blending ratio, 1:2; temperature, 120 °C; biomass loading, 20%; reaction time, 10 min.

Fundamentally, the catalyst plays a key role in promoting the decomposition of biomass and improving liquefaction efficiency. Among the reported catalysts, sulfuric acid was recognized as the best catalyst due to its excellent catalytic performance, resulting in high biomass conversion [15,30]. Aiming to optimize the acid catalyst loading for acetone-soluble lignin liquefaction, various contents of sulfuric acid, from 0 to 4% (based on solvent weight), were applied. The corresponding biomass conversion as a function of catalyst loading with respect to acid and hydroxyl numbers of biopolyol is presented in Figure 4. It can be observed that the biomass conversion was significantly improved when 1% catalyst loading was used compared to that of noncatalyzed liquefaction. Meanwhile, the continued increase in catalyst loading led to a gradual reduction in conversion and the highest biomass conversion was 69.18% at 1% catalyst loading. The observed decrease in biomass conversion with high catalyst loading might be due to the accelerated detrimental recondensation reaction between the liquefied compounds [2,15,31]. Additionally, at 1% catalyst loading, produced biopolyol exhibited a low acid number and a reasonable hydroxyl number. Therefore, the optimal loading catalyst was selected as 1%, with which the conversion was maximized and the properties of biopolyol were suitable for later biopolyurethane synthesis. The optimal 1% catalyst loading in this work was more promising and significant compared to the common acid loading of around 3–4%, as previously reported [15]. Furthermore, using low catalyst loading at 1% could reduce the cost of raw materials for biopolyol production as well as restrict the possible corrosion of metal equipment used in the process.

#### 3.2.4. Effect of Reaction Temperature

**Figure 5:**
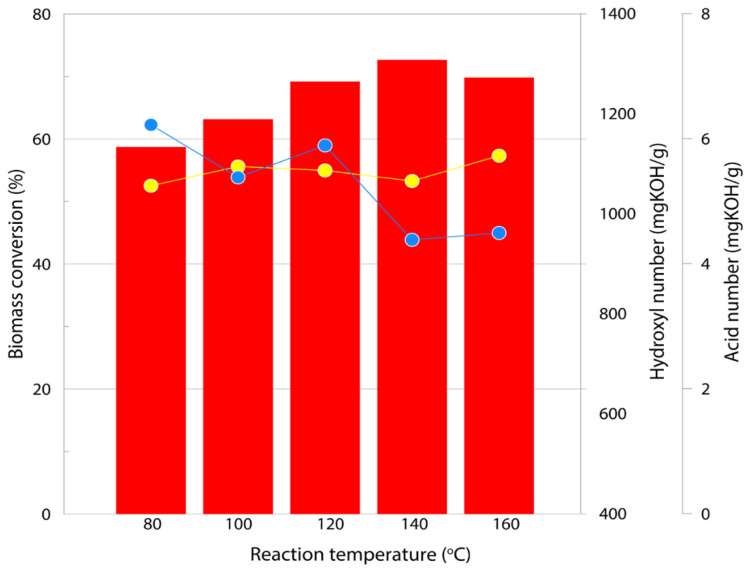
Effect of reaction temperature on biomass conversion and biopolyol acid number (blue line) and hydroxyl number (yellow line). Reaction condition: solvent blending ratio, 1:2; biomass loading, 20%; catalyst loading, 1%; reaction time, 10 min.

Liquefaction of lignocellulosic biomass is usually conducted in the temperature range of 130–250 °C, depending on the chosen catalyst. In general, base-catalyzed liquefaction requires high temperatures around 250 °C to achieve high biomass conversion, while acid-catalyzed liquefaction needs a lower reaction temperature [15,17]. With the application of microwave heating, due to the direct heat transfer at the molecular level, the reaction temperature is believed to be reduced with no considerable change in conversion [24]. Therefore, in this study, a range of mild temperatures from 80 °C to 160 °C was applied to determine the optimal condition. From Figure 5, we observed that the biomass conversion increased with the rising temperature, from 58.71% at 80 °C to 72.64% at 140 °C. A continual increase in reaction temperature to 160 °C significantly reduced the conversion of acetone-soluble lignin to biopolyol to 69.81%. The reduction of acetone-soluble lignin conversion at high temperatures is mainly due to the recondensation between liquefied products and the side reactions of remaining impurities in the unrefined crude glycerol solvent, which might result in residue formation [31]. Moreover, a low acid number and a reasonable hydroxyl number of biopolyol obtained from the 140 °C liquefaction condition were suitable for biopolyurethane production. Therefore, the optimal reaction temperature for acetone-soluble lignin microwave-assisted liquefaction was selected as 140 °C. Based on the aforementioned results, the optimal reaction condition for microwave-assisted two-step liquefaction of acetone-soluble lignin was determined at a 1:2 blending ratio of CG to BD, 20% biomass loading, 1% catalyst loading, 140 °C reaction temperature, and a fixed reaction time of 10 min.

### 3.3. Characterization of Acetone-Soluble Lignin, Biopolyol, and Synthesized Polyurethane Foams

#### 3.3.1. FT-IR Analysis

**Figure 6:**
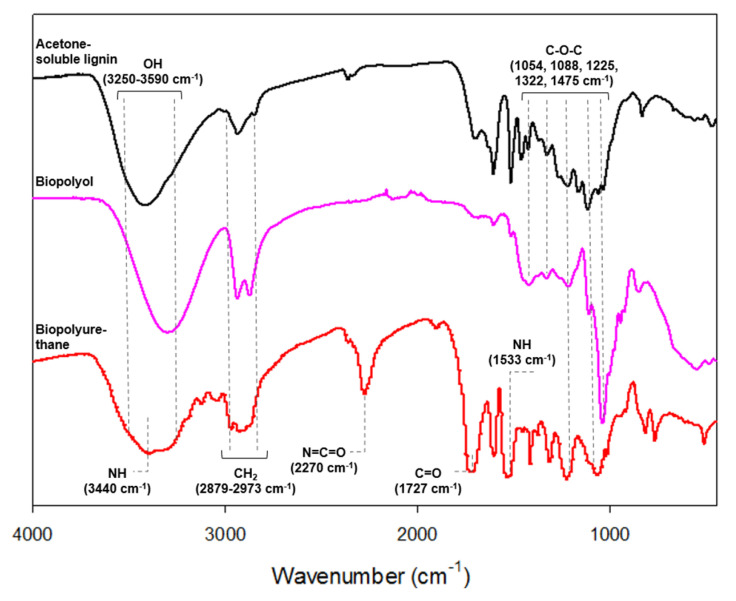
FT-IR spectra of acetone-soluble lignin, optimal biopolyol, and biopolyurethane foam.

**Figure 7:**
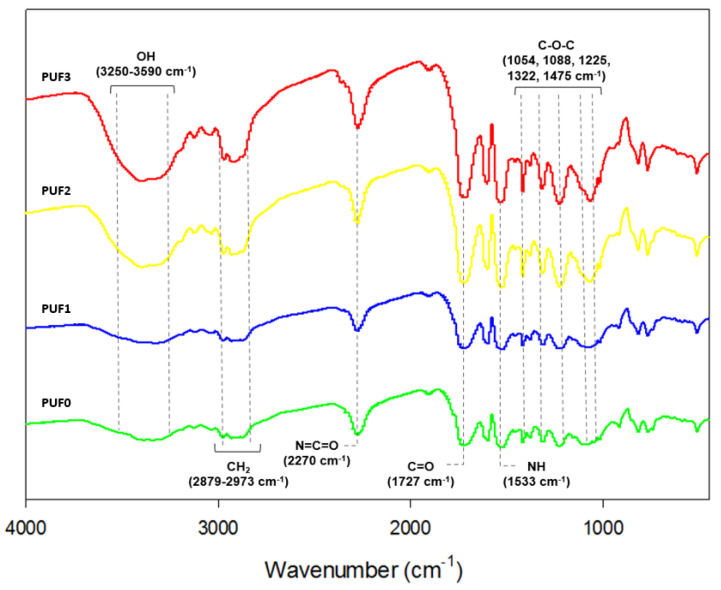
FT-IR spectra of neat polyurethane foam and biopolyurethane foams.

In terms of characterization of biomass, biopolyol, and biopolyurethane, FT-IR analysis is one of the most common technologies for studying the chemical structure and confirming the functional groups present in a polymer matrix. FT-IR spectra of acetone-soluble lignin, biopolyol, neat polyurethane foam, and biopolyurethane foams were recorded and are shown in Figure 6 and Figure 7. As mentioned above, the main functional group of acetone-soluble lignin is the hydroxyl group, which could refer to the wideband between 3200–3600 cm^−1^ [18,32]. Additionally, the presence of this band in the biopolyol spectrum indicated that the liquefaction of acetone-soluble lignin was successful and the resultant biopolyol was suitable for the production of biopolyurethane foam. In addition, the peaks at 2879–2973 cm^−1^ in all samples could be assigned to the stretching vibration of the –CH_2_ groups. Meanwhile, the peaks at 1054 cm^−1^, 1088 cm^−1^, 1225 cm^−1^, 1322 cm^−1^, and 1475 cm^−1^ mainly represented –C–O–C bonds in the structure of acetone-soluble lignin, biopolyol, and biopolyurethane foam [29]. Compared to acetone-soluble lignin and biopolyol, some new peaks were observed in the biopolyurethane spectrum at 1533 cm^−1^ and 1727 cm^−1^. These peaks were, respectively, attributed to the –NH bond and –C=O bond, which confirmed the formation of urethane linkages after polyurethane synthesis [19]. However, it can be seen in Figure 7 that the –NCO and –OH bands were still present in the spectra of polyurethane foams after polymerization at 2270 cm^−1^ and 3250–3590 cm^−1^, respectively. Accordingly, FT-IR spectra of PUF0 and PUF1 were quite similar and the free –NCO and –OH bands had low intensity. This result indicated that the polymerization was almost completed because most of the reactants were reacted and converted to polymer foams. In the case of PUF2 and PUF3, even the presence of new peaks related to urethane linkage was observed, and there were still relatively high-intensity peaks of free –OH and –NCO. Due to the high hydroxyl number and irregular structure of biopolyol compared to the homogeneous and well-defined structure of commercial polyol, the addition of more biopolyol involved in the polymerization might result in fast reactions in the blowing process and a rapid foaming time. These rapid processes subsequently prevented total consumption of bound –NCO and –OH groups because they are locked in the foam matrix [33]. The exact mechanism of why there were still high peaks of free –OH and –NCO needs to be investigated. For further determining the best biopolyurethane foam, the thermal and physical properties of polyurethane foams need to be also analyzed to confirm whether they could be used as a promising renewable alternative to the traditional petroleum-based polyurethane foam.

#### 3.3.2. Thermogravimetric Analysis

**Figure 8:**
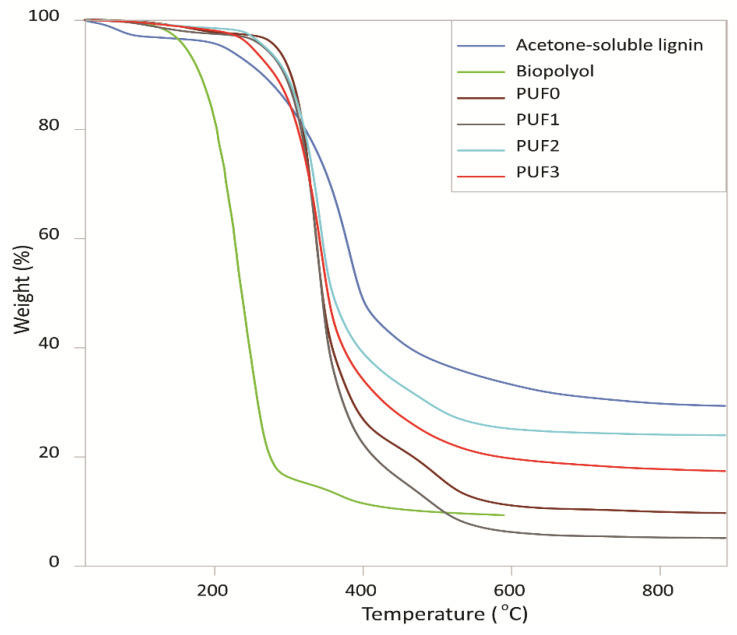
TGA curves of acetone-soluble lignin, optimal biopolyol, synthesized polyurethane foams.

**Figure 9:**
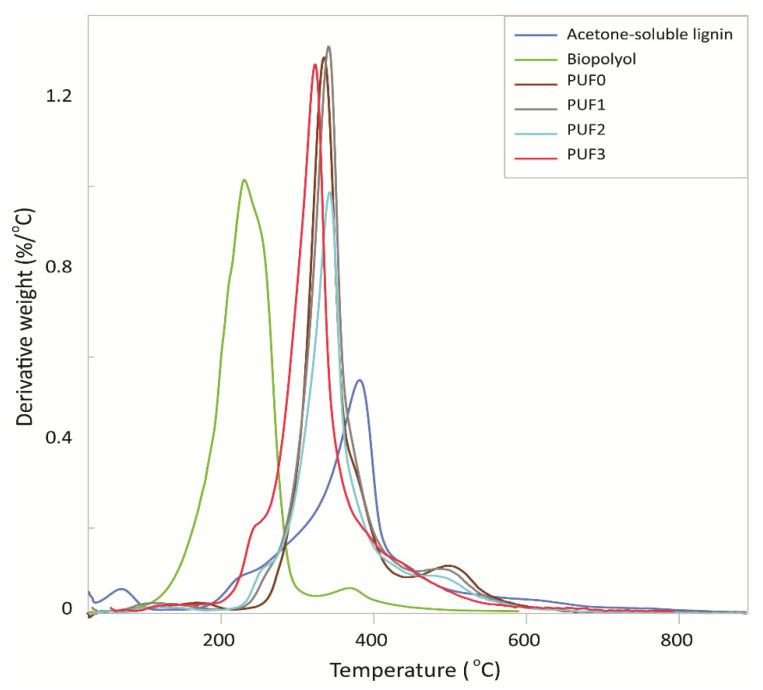
DTG curves of acetone-soluble lignin, optimal biopolyol, synthesized polyurethane foams.

In the present study, thermogravimetric analysis was conducted to assess the thermal properties of acetone-soluble lignin, biopolyol obtained at the optimal liquefaction condition, and produced polyurethane foams. Accordingly, Figure 8 and Figure 9 show the TGA curves and DTG curves of produce polyurethane foams, respectively, and typical thermal properties are presented in Table 2. With acetone-soluble lignin, it can be observed in Figure 8 that there were three main stages of weight loss. The initial stage occurred below 100 °C, which could be attributed to the evaporation of the remaining moisture in acetone-soluble lignin. The second weight loss region took place at 200–300 °C, representing the degradation of hemicellulose, while the major weight loss in the temperature range 300–500 °C was mainly due to the degradation of cellulose and lignin [34]. In contrast to acetone-soluble lignin, biopolyol only showed two regions of weight loss at around 150–400 °C due to the complete elimination of moisture after liquefaction.

Fundamentally, polyurethane is reported as a thermally unstable material due to the presence of urethane linkages [16]. As shown in Figure 8, all polyurethane foams had a similar shape in their weight loss curves, indicating similar thermal behaviors. Specifically, there were two distinct areas of weight loss for all polyurethane samples. The first degradation stage that occurred at 250–400 °C could be assigned to the breakdown of urethane bonds, while the small peak at 400–550 °C was due to the decomposition of unreacted polyols or isocyanates [14]. Based on Table 2, some thermal properties of biopolyurethane foams, such as the temperature at 5% weight loss and 10% weight loss, were slightly lower than that of the neat foam. However, when the temperature increased to 350 °C, PUF2 and PUF3 exhibited better stability than neat foam PUF0. In general, it can be stated that lignin-based biopolyurethane had comparable thermal properties sufficient for a wide range of applications in packaging, construction, or insulation, similar to petroleum-based polyurethane foam.

#### 3.3.3. Determination of Density and Compressive Strength

**Figure 10:**
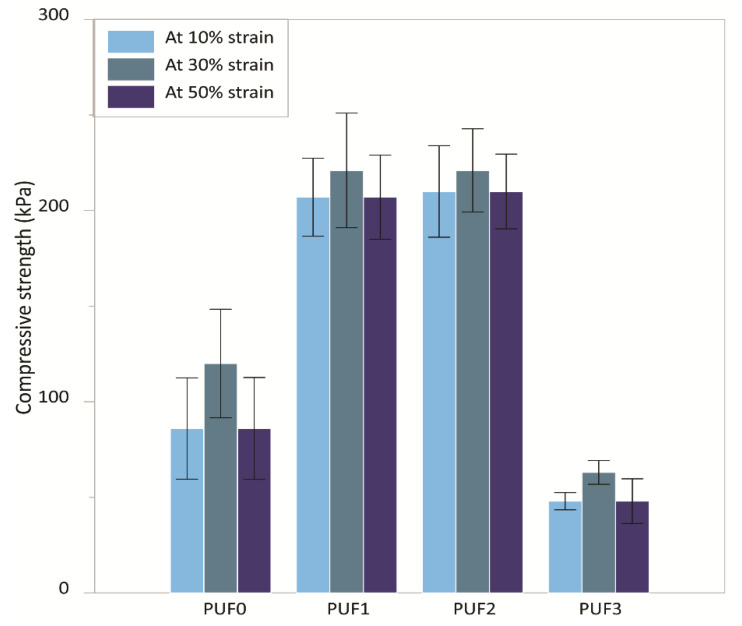
Compressive strength of synthesized polyurethane foams.

**Figure 11:**
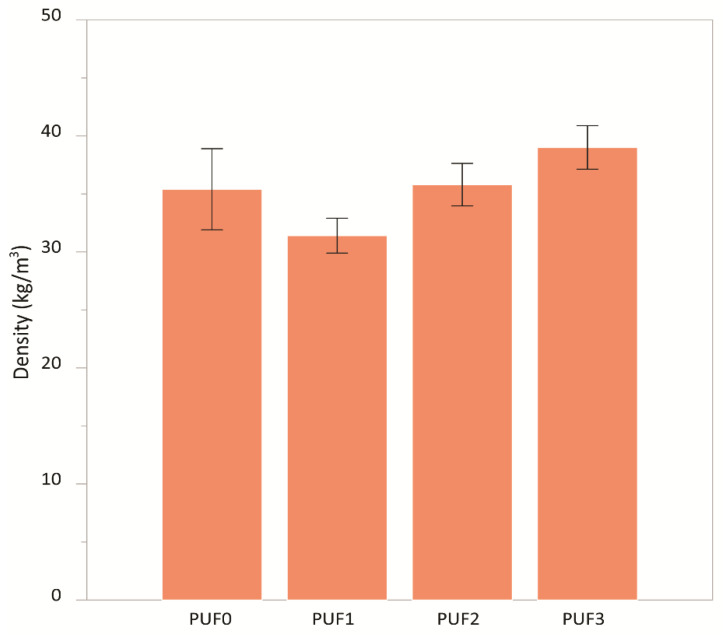
Density of synthesized polyurethane foams.

Mechanical properties including compressive strength and density are important factors for identifying the suitable applications of polyurethane foam. In this study, various biopolyol contents were applied for biopolyurethane synthesis and the mechanical properties of all foams were comprehensively determined. As shown in Figure 10 and Figure 11, biopolyurethane foams PUF1 and PUF2 possessed relatively similar density but significantly better compressive strength compared to the neat foam PUF0. These improvements in the mechanical properties of biopolyurethane foams PUF1 and PUF2 are probably due to the increase in hard segment contents and crosslinking densities in the polyurethane network [16]. However, when increasing the content of biopolyol in the polyol mixture to 30%, the large content of the hard segment caused a dramatic decrease in compressive strength of PUF3 to 48 kPa and 63 kPa at 10% strain and 30% strain, respectively. In this case, the high content of the hard segment might reduce the uniform distribution in the polymer matrix, resulting in a weakened strength of polyurethane foam. Combining both mechanical properties and the objective to ensure the techno-economic feasibility of biopolyurethane production from acetone-soluble lignin of silvergrass saccharification residue, PUF2 was selected as the best biopolyurethane foam in terms of the predominant properties compared to petroleum-based polyurethane foam. With a density of 35.8 kg/m^3^ and a compressive strength of around 210–221 kPa, biopolyurethane foam PUF2 can be used as a low-density foam, which is highly favorable for diverse applications in packaging, construction, and insulation.

#### 3.3.4. Determination of Cell Morphology of Polyurethane Foams by HR-SEM

**Figure 12:**
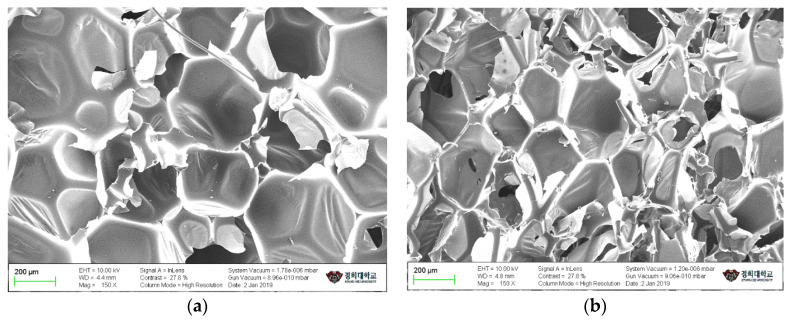
Cell morphology structure of neat polyurethane foam (**a**) and biopolyurethane foam (**b**).

**Figure 13:**
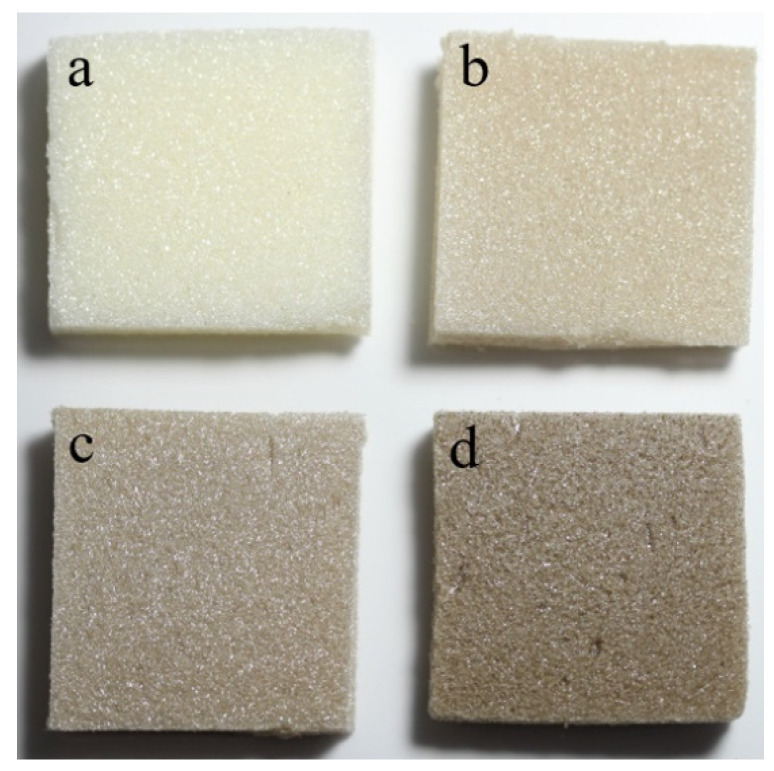
Photographs of synthesized polyurethane foams PUF0 (**a**), PUF1 (**b**), PUF2 (**c**), and PUF3 (**d**).

In addition to thermal properties and mechanical properties, the cell structure and morphology of the produced foams were also evaluated. SEM images of both the neat foam PUF0 and biopolyurethane foam PUF2 were recorded and are shown in Figure 12. Compared to the neat foam, biopolyurethane foam displayed a relatively small change in cell morphology. In general, all foams showed a closed-cell structure with similar cell wall thicknesses. These results indicated that the biopolyol was well dispersed in the polyol mixture and the resultant polyurethane linkages inside the polymer matrix were well organized [35]. However, Figure 12 shows that biopolyurethane foam had a smaller pore diameter than the neat foam. The average pore diameter of the biopolyurethane foam containing 20% of biopolyol and the neat polyurethane foam was approximately 250 μm and 350 μm, respectively. As previously reported, the introduction of biopolyol in polyurethane foam production as a substitute for polyol might prolong the polymerization reaction and break the equilibrium between the foaming and gelling reaction in polymerization, resulting in a smaller pore structure [36]. Although there was a small change in cell morphology when using biopolyol for polyurethane preparation, the uniform distribution of pore size and the reasonable cell wall thickness of biopolyurethane foam still made it a promising candidate for the replacement of petroleum-based polymers. For visualization, photographs of synthesized polyurethane foams are presented in Figure 13. The difference in color of produced foams is attributed to the different biopolyol contents involved in the polymerization reaction.

## 4. Conclusions

A high-lignin-containing acetone-soluble fraction of silvergrass saccharification residue was obtained from *Miscanthus sacchariflorus*, and then successfully liquefied by microwave-assisted two-step liquefaction using a crude glycerol and 1,4-butanediol solvent mixture. To optimize the condition for acid-catalyzed liquefaction of acetone-soluble lignin of silvergrass saccharification residue, different solvent blending ratios, biomass loadings, catalyst loadings, and reaction temperatures were employed. The optimal condition was determined at a 1:2 of crude glycerol to 1,4-butanediol ratio, 20% biomass loading, 1% catalyst loading, and 140 °C reaction temperature. In this condition, 72.64% biomass conversion was achieved after only 10 min of reaction and the produced biopolyol had a 4.38 mg KOH/g acid number. The obtained results proved that the utilization of microwave heating remarkably facilitated the efficient two-step liquefaction of lignin to produce high quality biopolyol. From the optimal biopolyol, a series of biopolyurethane foams with high performance was successfully produced. The chemical structure, thermal stability, mechanical properties, and cell structure of biopolyurethane foam and petroleum-based polyurethane foam were comprehensively analyzed. The results showed that the biopolyurethane foam exhibited comparable thermal behaviors, similar foam structure, and even better compressive strength compared to that of petroleum-based polyurethane foam. In conclusion, acetone-soluble lignin of silvergrass saccharification residue is a promising renewable feedstock for the production of biopolyol and biopolymers such as biopolyurethane foam.

## Figures and Tables

**Table 1:** Composition of *Miscanthus* feedstock, pretreatment solid, and saccharification residue.

Component	Composition (g/100 g Dry Biomass)		
	Feedstock	Pretreatment Solid	Saccharification Residue
Cellulose	37.4 ± 0.0	57.2 ± 0.5	12.4 ± 0.0
Hemicelluloses	26.5 ± 0.1	4.0 ± 0.1	1.9 ± 0.0
Klason lignin	25.1 ± 0.0	28.3 ± 0.9	39.2 ± 0.2
Water extractives	9.7 ± 0.4 (2.2 g glucose and others)	1.9 ± 0.2 (0.1 g glucose, 1.3 g xylose, 0.1 g arabinose, 0.3 g acetic acid)	6.8 ± 0.1 (2.3 g glucose, 0.4 g xylose, 0.1 g arabinose, 0.7 g protein, 1.2 g phenolics, 2.1 g unknown)
Ethanol extractives	1.4 ± 0.0	Not measured	31.6 ± 1.3
Ash	3.1 ± 0.0	1.9 ± 0.1	5.7 ± 0.1

**Table 2:** Thermal properties of acetone-soluble lignin of silvergrass saccharification residue, optimal biopolyol, and synthesized polyurethane foams.

Sample	Temperature at 5% Weight Loss (T_d5_)	Temperature at 10% Weight Loss (T_d10_)	Char Yield (%)
Acetone-soluble lignin	215.62	262.50	34.54
Biopolyol	164.06	181.25	9.09
PUF0	287.50	306.25	10.91
PUF1	265.62	292.19	6.36
PUF2	266.12	293.05	25.54
PUF3	253.12	281.25	20.00

## Data Availability

Data is contained within this article.
